# Editorial: Underlying molecular interconnections of the estrogen receptor alpha and associated factors involved in breast cancer development: The way to new therapeutic approaches

**DOI:** 10.3389/fendo.2022.1094711

**Published:** 2022-12-20

**Authors:** Guy Leclercq, Filippo Acconcia

**Affiliations:** ^1^ Institute Jules Bordet, Cancer Center of the Université Libre de Bruxelles, Brussels, Belgium; ^2^ Department of Science, University Roma TRE, Rome, Italy

**Keywords:** estrogen, receptor,breast cancer, therapy, ER alpha, GPR30

Estrogen receptors (ER)α and β contribute to the evolution of hormone-dependent breast cancers, an evolution that may be counteracted by antiestrogens or aromatase inhibitors which block estrogen synthesis. Implication of ERα in this therapy justifies its selection for this publication, although ERβ may contribute through specific antagonist activity.

ERα (as all steroid hormone receptors) belongs to the family of “nuclear receptors”, which modulate the expression of genes working as transcription factors. The localization of a large majority of the active receptors within the cell nucleus justifies this classification terminology, even if a palmitoylated receptor form anchored at the plasma membrane operates by inducing transient signal transduction pathways. In fact, both receptor pools usually cooperate, the membrane receptor acting largely before the nuclear form to generate coherent and irreversible transcriptions to maintain homeostasis or induce evolution to satisfy extra- and intra-cellular requirements. In this context, a complementary G protein coupled-estrogen receptor structurally distinct from ERα localized within the plasma membrane—GPR30—should be mentioned, as it interacts with a “coregulator” recruitment hub of ERα (aa 295-311). The presence of GPR30 together with a truncated receptor form (ERα36) present in ERα-negative cells opens possibilities for new therapeutic approaches especially devoted to endocrine-resistant cancers. Acramel and Jacquot provide recent data concerning this topic.

An early step of steroid hormone receptor-mediated transcriptions is the interruption of repressive action exerted by chaperones, especially Hsp90. This liberation allows the exposure of nuclear localization signals (NLS) of the receptors required for their nuclear anchorage. The recruitment of importins at the level of these NLSs contributes to the underlying transfer, as overviewed by Kalyvianaki et al. With regard to ERα, importin α seems to be of considerable importance. The presence of a recruitment site for the latter within the hinge region of this receptor (aa 266-269) in the proximity of the aa 295-311 co-hub suggests a coordinative action of this importin and coregulators to induce ERα structural changes required for transcription. Calmodulin complex, which occurs at this level, must be mentioned, since it stabilizes the receptor in a homo-dimeric form required for large parts of its transcriptions.

The ERα homo-dimeric form induces the expression of genes under the control of a DNA palindromic motif (Estrogen Response Element) with which it associates, while its hetero-dimeric form refers to a complex with another transcription factor bound to its own response element. Hence, the homo-dimer directs the whole transcription procedure while the hetero-dimer assists the action of a partner. Both mechanisms implying successive transient recruitment of a myriad of coregulators for the progression of the transcription cycle. Enzymatic capacity of a set of such co-regulators (kinases, phosphatases, methylases, acetylases, ubiquitin ligases) confers to this cycle an irreversible process which impacts the expression of genes implicated in the proliferation and evolution of the tumors and their ERα levels. Tecalco-Cruz et al. extensively overviews the underlying mechanisms of this regulation.

The existence of a reverse relationship between the levels of ERα and Ki67, a marker of proliferation and histological grading of malignancy (giving rise to an ERα-negative/high Ki67 status at G3), suggests a connection between the ERα turnover rate and cell growth cycle. Interplay between ERα and cyclin D1 supports this view: the receptor promoting the expression of the CCND1 gene enhances proliferation and therefore tumor evolution. Since CCND1 overexpression is common in ERα-positive breast cancers unresponsive to endocrine treatments, this amplification has been suggested as being responsible at least in part for this resistance. A meta-analysis reported by Jeffreys et al. of 6,400 women with ERα-positive post-menopausal breast cancer of which 18% displayed an overexpression of CCND1 provides data in agreement with this view. Even though disparities between data were taken into account, CCNDN1 amplification emerged as a significant prognostic index of reduced recurrence-free interval and overall survival.

Previous studies refer to ERα-positive breast cancers of which evolution may be counteracted by an adjuvant postsurgical tamoxifen therapy. If resistance unfortunately occurs, it may be interrupted by high-dose “linear” strong estrogens (E2,DES). *V.C. Jordan et al.* demonstrated that this salvatory effect is connected to an overactivated ERα transcription of proteins inducing apoptosis (especially AP-1 family members). At the level of the endoplasmic reticulum, these proteins provoke various stress responses with lethal potency, a kinase (PERK) playing a role of major importance. Maximov et al. provides an overview of this topic, showing a complementary structure-activity relationship relative to a set of non-linear weak estrogens to assess whether these compounds may similarly induce apoptosis. Their ability to localize Helix-12 of the ligand-binding site of ERα in an agonist position unfavorable for major proliferation enhancement without affecting apoptosis induction makes possible a curative therapy devoid of the deleterious side-effects recorded with E2 and DES. If this can be confirmed, future research should investigate whether this approach could be extended to synthetic peptides interfering with coregulator recruitment. ERα17p (corresponding to the 295-311 aa hub), which stimulates or inhibits the development of tumors depending on the experimental protocols used, could be suitable to conduct such a study.

In another therapeutic pilot, an association of E2 with progesterone and other progestins was investigated by Perkins et al. in view of reports suggesting a potential utility of a combination of ER/PR ligands for breast cancer treatment. All tested progestins promoted breast cancer cell proliferation, albeit to different extents, through a mechanism requiring an association of ERα and PR on the Myc promoter. This finding, in the absence of complementary data, underscores the potential for such an ERα/PR crosstalk ligand-based therapy. The assessment of antagonistic activity through putative ERβ induction should be investigated.

*The six investigations mentioned here represent just a few facets of our publication project. This justifies the topic to be pursued in a future volume. Hence, publication submissions are still welcome.*

## Author contributions

All authors listed have made a substantial, direct, and intellectual contribution to the work and approved it for publication.

